# Immunoglobulin Light Chain (AL) Amyloidosis Preceding Marginal Zone Lymphoma: A Case Report

**DOI:** 10.7759/cureus.26517

**Published:** 2022-07-02

**Authors:** Krishna Doshi, Jacob Bitran, Brian Adley, Nahren Asado

**Affiliations:** 1 Internal Medicine, Advocate Lutheran General Hospital, Park Ridge, USA; 2 Hematology and Oncology, Advocate Lutheran General Hospital, Park Ridge, USA; 3 Pathology, Advocate Lutheran General Hospital, Park Ridge, USA

**Keywords:** b-cell lymphoma, bortezomib, congo red, hematology-oncology, marginal zone lymphoma mzl, al amyloidosis

## Abstract

Immunoglobulin light chain (AL) amyloidosis is a systemic disease in which different systems such as kidneys, heart, and lungs are affected by the deposition of amyloid, a form of fibrillary protein. Usually, it occurs in patients with pre-existing diagnoses of plasma cell dyscrasias and is rarely seen in the concurrence of marginal zone lymphoma (MZL). Earlier interventions with cyclophosphamide and dexamethasone in conjunction with newer therapies such as bortezomib, carfilzomib or lenalidomide, and pomalidomide are being used to treat patients with AL amyloidosis. In this report, we are presenting a unique case of a patient who was diagnosed with AL amyloidosis several years prior to presenting with a soft tissue mass, which was subsequently noted to be an amyloid mass within an MZL. Overall, the occurrence of AL amyloidosis and MZL is rare with less than 20 patients reported. The MZL developed prior to or simultaneously with AL amyloidosis in the reported cases. Therefore, to our knowledge, this is the first time systemic amyloidosis has preceded MZL.

## Introduction

Amyloidosis is characterized by the abnormal tissue deposition of fibrillary protein aggregates, typically light chains in beta-pleated sheets [1,2]. Amyloidosis is subtyped by being either primary (AL), secondary (AA), or familial (transthyretin amyloidosis (ATTR)) [1,2]. Immunoglobulin light chain (AL) amyloidosis is a lymphoproliferative disorder of plasma cells that produces immunoglobulin light chains (typically kappa), resulting in amyloid deposition in organ systems such as the heart or kidneys that can severely impair cardiac or renal function. The incidence rate of AL amyloidosis increases in each decade of life after 40 years, with the median age of diagnosis being 64 years [1]. AL amyloidosis can commonly occur and lead to complications. Amyloidosis rarely occurs in patients with low-grade lymphoproliferative disorders such as mucosal-associated marginal zone lymphoma (MALT) and even more rarely in patients with marginal zone lymphoma (MZL) without mucosal involvement.

Amyloid complicating MALT typically is localized (lung, skin, and lymph nodes), and systemic amyloidosis is unlikely [3-6]. Stuhlmann-Laeisz et al. reported that in 14/21 patients with MALT-associated amyloidosis, MALT preceded the diagnosis of amyloid by a median of 64 months; in the remaining seven patients, the diagnosis of MALT and amyloid was made concurrently. Telio et al. reported two distinct syndromes of lymphoma associated with AL amyloidosis. One pattern is peritumoral, which is associated with MALT. The second is systemic amyloidosis, which is associated with Waldenstrom’s macroglobulinemia and carries a poor prognosis [7]. Amyloidosis complicating MZL is rare, and systemic renal amyloidosis complicating MZL is even rarer. Standard treatments for AL amyloidosis include melphalan and prednisolone or cyclophosphamide and dexamethasone. Newer therapies are now available for treatment options, such as bortezomib, which rapidly reduces the serum-free light chain concentration in patients, and due to its efficacy in achieving hematological and organ response, it is the frontline therapy in AL amyloidosis [8]. We are presenting a patient diagnosed with systemic amyloidosis with renal involvement 11 years prior to the diagnosis of MZL.

## Case presentation

**Figure 1:**
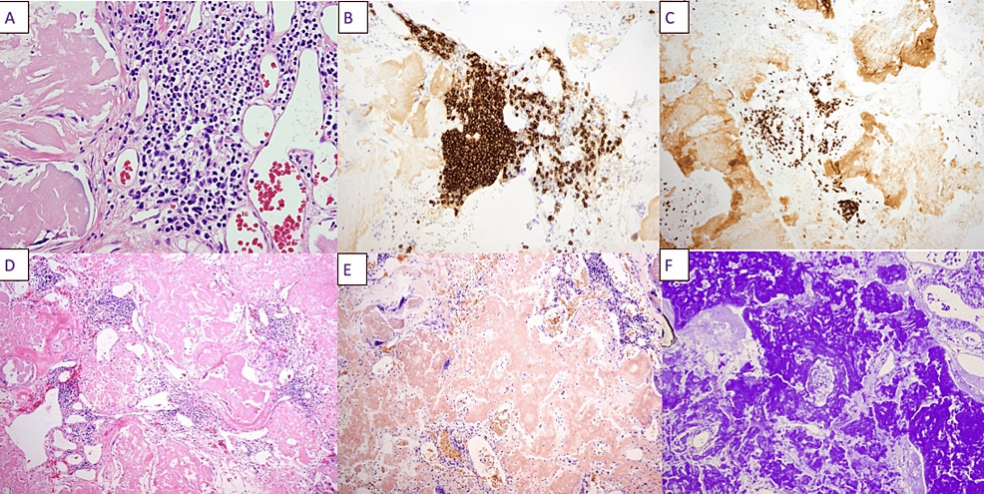
Pathology slides of the left groin mass. A: Hematoxylin and eosin (H&E) stain showing the aggregate of lymphoid cells, which are small in size with moderate pale cytoplasm associated with some plasma cells (40x). B: CD20 cells. C: CD5 cells. D: H&E stain low power image showing multiple lymphoid aggregates associated with extensive areas of pink amorphous martial deposition (10x). E: Congo red under light microscopy showing positive brick red staining of the abnormal material. Congo red under polarized light showing apple-green birefringence consistent with amyloidosis (10x). F: Abnormal material stain positive with crystal violet (20x).

The patient was a 54-year-old female with a past medical history of AL amyloidosis with kappa-restricted plasma cells initially diagnosed in 2011, 11 years prior to the current presentation, on the basis of a soft tissue biopsy. During the time of her diagnosis, 11 years prior, she had no evidence of cardiac involvement based on a cardiac MRI and her renal function was normal. She was advised to undergo autologous bone marrow transplantation, which she declined. She was lost to follow-up and did not receive medical care until November 2020, when she presented to an outside hospital with acute renal failure; her serum creatinine was 14 mg/dL (baseline: unknown). She was diagnosed with systemic amyloidosis with renal involvement and was started on hemodialysis. On December 2, 2020, she was started on chemotherapy with cyclophosphamide, DNA alkylating agent, bortezomib, which rapidly reduces serum-free light chains, and dexamethasone (CyBord). She was transferred from the outside hospital for management of septic arthritis and evaluation of a 17-cm mass in the left groin. Septic arthritis was treated with intravenous vancomycin on M/W/F, dosed per renal function. General surgery and plastic surgery were consulted for resection of the left groin mass. The soft tissue proved to contain amyloid, which was confirmed by Congo red and crystal violet stains. There was an infiltrate of small lymphocytes positive for CD20 and CD79a, consistent with MZL (Figure 1). Following wound healing, the patient was started on daratumumab, a monoclonal antibody targeting plasma cell antigen CD38, bortezomib, and dexamethasone, which she continues to the present time on an ambulatory basis.

## Discussion

AL amyloidosis can be associated with B cell neoplasia, including MALT, chronic lymphatic leukemia, and Waldenstrom’s macroglobulinemia [6-9]. The association of MZL with AL amyloidosis is rare, and a handful of cases have been reported [6].

Our patient is unique for several reasons. First, the diagnosis of AL amyloidosis preceded that of lymphoma by several years. Current literature shows that lymphoma usually precedes AL amyloidosis. One study showed that in 14 out of 21 patients with systemic AL amyloidosis, the diagnosis of lymphoma preceded the clinical manifestation of amyloidosis by a median of 64 months [3]. This case is unusual as the diagnosis of systemic AL amyloidosis preceded that of lymphoma by several years. Second, this patient responded well to the therapies, which is unusual, and has had an extended survival response since receiving treatment. As noted in one study, the median survival from the diagnosis of systemic amyloidosis in patients with nonlymphoblastic lymphoproliferative disorders was 26 months [5]. In our case, the patient was diagnosed with AL amyloidosis several years prior and then developed a mass that subsequently demonstrated Congo red stain in tissue in concurrence with B cells, which is rare. One could argue that MZL may have been present in our patient in 2011 in a subclinical manner. However, it is highly unlikely that MZL would be clinically quiescent for nine years.

One aspect that deserves investigation is whether the AL amyloidosis and MZL arose from a single pre-B cell or B cell clone or represent clonal differentiation of a B cell progenitor among two allied by divergent pathways. The AL amyloid plasma cell produced kappa light chains. The MZL expressed both kappa and lambda light chains suggestive of differentiation along divergent pathways. Saltman et al. and Fermand et al. have documented a single cell of origin of chronic lymphocytic leukemia (CLL) associated with myeloma [10-12]. While this may be the case in CLL with myeloma, the alternate explanation is a common B cell progenitor differentiating into two allied but different pathways.

## Conclusions

In conclusion, we described a rare case with AL amyloidosis preceding MZL by several years. Moreover, MZL should be kept as a differential diagnosis for patients with AL amyloidosis and a new soft tissue mass to initiate suitable treatment in appropriate clinical settings.
